# Innovative multi-protectoral approach increases survival rate after vitrification of ovarian tissue and isolated follicles with improved results in comparison with conventional method

**DOI:** 10.1186/s13048-018-0437-5

**Published:** 2018-08-07

**Authors:** Dmitry Nikiforov, Valentina Russo, Delia Nardinocchi, Nicola Bernabò, Mauro Mattioli, Barbara Barboni

**Affiliations:** 1 Faculty of Bioscience, Unit of Basic and Applied Biosciences, University of Teramo, 64100, via R. Balzarini 1, Teramo, Italy; 2 Istituto Zooprofilattico Sperimentale dell’Abruzzo e del Molise “Giuseppe Caporale” (IZSAM), 64100 Teramo, Italy

**Keywords:** Vitrification, Slow freezing, Ovarian tissue, Isolated follicles, Cryopreservation, Fertility preservation, Ovary

## Abstract

**Background:**

In recent years, autotransplantation of cryopreserved ovarian tissue became a promising approach to preserve female fertility. The slow freezing is the most effective technique which resulted in greater live birth incidence so far. Despite that, interest to vitrification of the ovarian tissue is swiftly growing, thereby undermining the necessity for further improvements in the technique. In present study, we evaluated possibilities to increase follicle survival rates adopting innovative multi-protectoral vitrification protocols, applied to the slivers of ovarian cortex or isolated early-antral follicles, frozen individually. These experimental protocols have been compared with with validated vitrification and slow freezing ones, clinically used for female fertility preservation.

**Results:**

The results showed that third tested variation of experimental vitrification protocol, with four cryoprotectants in relatively low concentrations and applied to pieces of ovarian tissue at 0 °C during equilibration, increased survival rate of ovine ovarian tissue and improved results in comparison with conventional vitrification method. This variation of experimental protocol showed significant increase in percentage of follicles with good morphology (69,3%) in comparison with only commercially available vitrification protocol for ovarian tissue (62,1%). Morphology results were confirmed by TUNEL assay. Analysis of estradiol and progesterone production by cultured individual follicles after freezing/thawing revealed that steroids secretion remained significantly higher after multi-protectoral vitrification and slow freezing protocol, when follicles after standard vitrification protocol demonstrated decline in steroidogenic activity.

**Conclusions:**

The multi-protectoral approach represents a workable solution to improve vitrification outcome on ovarian tissue and isolated follicles. The reduction of individual cryoprotectants concentrations, while maintaining their sufficient cumulative level in the final freezing solution, helps to increase efficiency of the procedure. Moreover, equilibration with lower temperatures helped to decrease even further the toxic effects of cryoprotectants and preserve original quality of ovarian tissue. Therefore, multi-protectoral vitrification can be suggested as an improved method for the clinical cryopreservation of ovarian tissue.

## Background

Chemo- and radiotherapy as well as bone marrow transplantation often lead to decline in ovarian reserve and have profound negative effects on female fertility [1]. The loss of reproductive endocrine support on hormonally sensitive tissues may lead to cascade of long-term diseases or syndromes reducing the quality of life of the patient [2]. Beyond cancer, several non-malignant diseases and conditions, as well as their treatments, can also negatively affect reproductive system [3]. Due to these factors, the oncofertility became fast developing direction in the reproductive medicine as it aims to ensure that recovered patients after gonadotoxic treatments could have a chance to conceive their own child.

Fertility preservation through cryoconservation of oocytes and embryos prior to gonadotoxic treatment represents a realistic option for patients who able to undergo an ovarian stimulation with further IVF procedure [4]. But there are groups of patients who can not have such a fertility solution: cancer patients with needs in urgent chemotherapy or prepubertal girls [5]. The only solution for these patients can be ovarian tissue cryopreservation. Such a technique followed by orthotopic reimplantation is considered as the most promising method to restore fertility in women who need to undergo immediate chemotherapy and prepubertal girls [6]. By the beggining of 2018, ovarian tissue cryopreservation resulted in at least 93 live births worldwide [7]. Ovarian cryopreservation can be performed by adopting two different methods: controlled slow freezing or vitrification [8]. The first one is well-established and validated to date for gametes and embryos for more than three decades [9–11]. Slow freezing was the first method adopted for ovarian tissue cryopreservation back in 1996 and was successfully in use ever since [12, 13]. Vitrification has not been proved yet as an advantageous method for ovarian tissue cryopreservation, but shall continue to be considered a promising technic as it proved to be secure method for cryopreservation of oocytes and preimplantation embryos [14–16]. Another prospective technique, which also aims to restore fertility for patients who need gonadotoxic treatment, is an in-vitro approach, when isolated early stage (pre-antral and early antral) follicles are being cultured in laboratory conditions with further in-vitro maturation of oocyte. Such technique is still considering as an experimental and not resulted in a live-birth yet.

Most of the live births after ovarian tissue cryopreservation so far have been achieved by controlled slow freezing [17]. Only two groups so far have reported live births from vitrification [18–20], while another centers around the world working with ovarian tissue cryopreservation still prefer slow freezing method. Pioneered protocol of slow freezing was developed in 1971 [21] and the first ever vitrification was performed in 1985 [22]. Despite tested efficacy of both methods for oocytes and embryos freezing, their further improvements remain desirable to decrease the associated iatrogenic and cryogenic damages, especially for the cryopreservation of ovarian tissue. Vitrification potentially can overcome negative effects of freezing by avoiding ice crystal formation. Another point of interest in vitrification is related to the low cost of this technology, which doesn’t require specific machines for freezing and can be implemented by trained staff in any laboratory.

During last decade, many studies have investigated vitrification outcomes on ovarian tissue or on isolated ovarian follicles of different animal models such as dogs [23], hamsters [24], rabbits [24], goats [25], monkeys [26], mice [27], rats [28] and ewes [29]. However, based on these reviews it is difficult to reach a conclusion on the success rate of vitrification, since the experimental data in these studies were frequently discrepant and results varied from one species to another. As a consequence, biological and technological conclusions on vitrification have not been drawn up yet. These conflicting results are probably due to the many variable aspects involved in vitrification protocols that vary between different groups, such as the size of ovarian tissue pieces, cryoprotectants used, exposure time to vitrification solutions or the carrier device for vitrification.

Present study was designed in purpose to explore if novel multi-protectoral vitrification protocol can help to overcome negative effects associated with cryopreservation. To evaluate the possibility of using of this experimental method for two described approaches on fertility restoration, we have done cryopreservation of both ovarian tissue slivers and isolated early-antral follicles and compared outcomes with two another established cryopreservation protocols.

## Methods

The present research was carried out by collecting sheep ovaries at the slaughterhouse from discarded tissues collected from animals intended for consumption. The present animal work has been conducted in strict accordance with relevant national and international guidelines. The authors have the permission of the Italian Ministry of Health to conduct animal experimentation according to the consent by silent contemplated by Italian law D.Lgs. 116/92, thus no permit/approval number was needed. The animals were not sacrificed and regained their production cycle. All chemicals were purchased from Sigma-Aldrich S.r.l. (Milan, Italy), unless other specified.

### Experimental design

The present study was designed to compare experimental multi-protectoral vitrification protocols with clinically validated ones and used so far for the ovarian tissue, such as slow freezing method [30] and the commercially available vitrification protocol (Kitazato, Japan) [31, 32]. Sheep ovaries were chosen as the experimental model as they have fibrous cortical tissue and relatively high primordial follicle density in the ovarian cortex similar to human ovaries. The multi-protectoral vitrification protocols, proposed in the present research, were designed to reduce the toxic effect of conventional cryoprotectants. Such reduction was achieved by creating cryopreservation solutions with four cryoprotectants in order to reduce their individual concentration, but maintaining cumulative concentration on the level sufficient for vitrification. The harmful toxic effect of cryoprotectants was also meant to be reduced by maintaining lower temperature (0 °C) during the equilibration procedure in order to slow down cellular metabolism prior to freezing procedure.

Starting from these premises, three cryopreservation protocols with different combinations of cryoprotectants were designed and compared with two techniques used clinically (Table 1). Their efficiency was evaluated by adopting morphological and immunofluorescent analyses as well as functional tests. For morphological evaluation ten cortical slivers of frozen/thawed ovarian tissue for each experimental group were analyzed. Immunofluorescent assay, aimed to detect DNA fragmentation (TUNEL technique), was performed by calculating the percentage of apoptotic follicles on the sections of the ovarian cortex. Finally, functional evaluation was performed by measuring estradiol and progesterone production during single early-antral follicles culture, which was carried out after isolated follicles were frozen and thawed individually. Steroids production was tested on 20 replicates of in-vitro cultured early-antral follicles for each experimental group.

**Table 1:** Summarised composition of the cryopreservation solutions used for described methods

Group	Protocol type	Cryoprotectants concentration					Reference	Experimental rational
		Penetrative			Non-penetrative			
		EG	PG	DMSO	Sucrose	Ficoll		
Protocol 1	Slow freezing	1,5 M	–	–	0,5 M	–	Schmidt et al. (2003) [30]	Well-known method established in clinical practise. No commercial media available
Protocol 2	Vitrification protocol (VIT 1)	–	3,6 M^a^	2,9 M^a^	0,5 M	–	Kagawa et al. (2007) [31]	Only commercially available vitrification medium for ovarian tissue resulted in a live birth to date
Protocol 3	Experimental vitrification protocol (VIT 2 with three variations)	1,5 M	2,5 M	–	1 M	10%	Experimental protocol	Experimental multi-protectoral vitrification protocol which combines four cryoprotectants at lower concentration on comparison with the protocol 2. DMSO was substituted with PG due to the DMSO’s higher cytotoxicity. The entire process of equilibration carried out on ice in purpose to slow down tissue metabolism
		2 M	2 M	–	1,5 M	10%		
		2,5 M	1,5 M	–	1 M	10%		

^a^highest concentrations used during the process in case for vitrification protocols

### Collection and preparation of ovarian tissue

Ovarian tissue was obtained from sheep pubertal ovaries, transported to the laboratory from the local abattoir in a thermo container within 1,5 h after collection. Upon arrival ovaries were separated from ligaments and rinsed in saline, contained 75 mg/L of penicillin-G and 50 mg/L of streptomycin sulphate. Then, the outer part of the ovarian surface was carefully isolated by the scalpel with the cortex thickness about 1 mm and medulla tissue was removed. Isolated ovarian cortex was finally cut into squares approximately 5*5 mm.

### Ovarian tissue cryopreservation

#### Protocol 1. Controlled slow freezing

The slow freezing protocol was carried out according to Schmidt et al. [30]. Briefly, tissue fragments were equilibrated for 30 min in 30 mL of cryomedium, containing 0,1 mol/L sucrose,1,5 mol/L of EG and 10 mg/mL of HSA in phosphate buffered solution on a tilting table on ice. Then, cortical fragments were transferred in Nunc CryoTubes (Nunc, Denmark) in 1 mL of the same cryomedium in a CryoLogic programmable temperature controller (CL-3300, CryoLogic, Australia) using Cryogenesis V5 software (Cryosolutions, US). The tubes were transferred to the set at 0 °C. Subsequently, the temperature was lowered at the rate of 2 °C/min until the temperature of − 9 °C was reached. At this stage, cryo tubes were seeded manually with a cotton swab dipped in liquid nitrogen. The temperature remained at − 9 °C for 5 more minutes. Next, at the rate of 0,3 °C/min the temperature decreased to − 40 °C and subsequently with 10 °C/min to − 120 °C before the vials were stored in liquid nitrogen.

For thawing vials were transferred to the 37 °C water bath until the ice inside vials disappeared and then pieces of cortex rinsed in 10 mL of three solutions (PBS containing 0,25 mol/L sucrose and 0,75 mol/L EG; then PBS containing only 0,25 mol/L sucrose; and PBS, respectively) for 10 min each under continuous agitation to wash out the cryoprotectants.

#### Protocol 2. Clinically validated vitrification protocol (VIT 1)

**Fig. 1:**
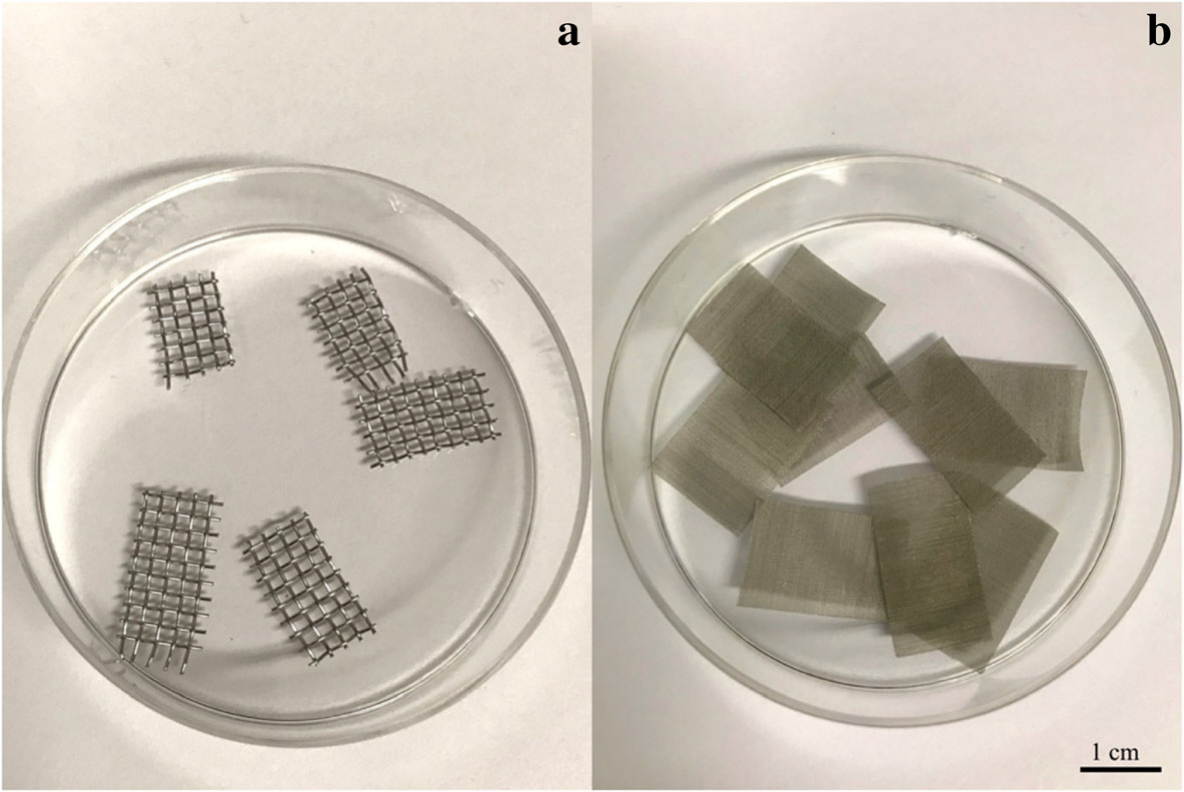
Stainless steel devises for carrying ovarian tissue slivers (**a**) or isolated follicles (**b**). Devices used for the vitrification procedure, made of the stainless steel wire with the diameters of 0,28 mm and 0,04 mm respectively

The protocol originally was described by Kagawa et al. [31, 32]. Ovarian tissue pieces were initially equilibrated in 1,3 mol/L of EG (corresponds to 7,5% *v*/v) and 1,1 mol/L of DMSO (corresponds to 7,5% *v*/v) in handling medium (HEPES-buffered TCM-199 solution supplemented with 20% serum substitute solution) for 25 min followed by a second equilibration in 3,6 mol/L (20% v/v) EG and 2,9 mol/L (20% v/v) DMSO with 0,5 mol/L sucrose for 15 min. Entire procedure was carried on at the room temperature. Ovarian tissue pieces then were placed in a minimum volume of solution onto a thin sterilized metal mesh with 1,31 mm aperture and 0,28 mm wire diameter (The mesh company, UK, Fig. 1, a), and submerged directly into liquid nitrogen, following which the mesh was inserted into a cryo vial and placed into a liquid nitrogen storage tank.

For warming, the mesh was removed from the vial and immersed directly into 40 mL of 37 °C handling medium supplemented with 1,0 mol/L sucrose for 1 min. Then, ovarian tissues were transferred into 15 mL of 0,5 mol/L sucrose in handling medium for 5 min at room temperature, and washed twice in pure handling medium for 10 min.

#### Protocol 3. Experimental vitrification protocol (VIT 2)

This protocol was developed using the multi-protectoral approach, combining two penetrative cryoprotectants: EG and PG with two non-penetrative: sucrose and ficoll. To determine the optimal combination of cryoprotectants, their three different combinations have been used (Table 1). Described concentrations were used to mix the stock solution, based on the handling medium (HEPES-buffered TCM-199 supplemented with 20% foetal bovine serum). Progressive exposition of the tissue to different solutions with increasing concentrations of cryoprotectants was introduced in purpose to allow stepwise diffusion of chemicals into the tissue. The stock solution was diluted as described further. For the first equilibration medium, incubation in which lasted 10 min and happened at room temperature, 25% of the stock solution used in the handling medium. Second equilibration (50% of the stock solution) was carried out on ice in order to decrease potential toxic effect of the cryomedium and lasted 10 min. Finally, the pieces of tissue were immersed into vitrification solution (stock) on ice for 7 min or until they were falling down to the bottom of the test-tube, in which they were equilibrated (on ice). Each equilibrating step was accompanied by consisting agitation. Ovarian tissue pieces then were placed in a minimum volume of solution onto a thin sterilized metal mesh (Fig. 1, a), and submerged directly into liquid nitrogen, following which the mesh was inserted into a cryo vial and placed into a liquid nitrogen storage tank.

For warming, the mesh was removed from the vial and immersed directly into 40 mL of 37 °C handling medium supplemented with 1,0 mol/L sucrose for 1 min. Then, ovarian tissue was transferred into 10 mL of 0,5; 0,25; 0,125 mol/L sucrose subsequently in handling medium for 5 min each step at room temperature and then washed twice in pure handling medium for 10 min.

### Histological evaluation of the cortical tissue

After freezing/thawing ovarian tissue pieces from the each experimental group and non-frozen tissue (control) were fixed overnight in a 4% paraformaldehyde and dehydrated in series of ethanol (70, 90, 95 and 100%). Absolute ethanol was replaced with xylene and then tissue was embedded in paraplast wax (Leica Bio Systems, Germany) at 56 °C.The samples were sectioned (5 μm) and mounted on polylysine slides, then allowed to dry overnight at room temperature before staining with haematoxylin and eosin. Sections have been analyzed with light microscopy and all found follicles were classified into two categories: good quality follicles and damaged/degenerated. First group characterized by oocytes without any contraction of cytoplasm or any pyknotic nucleus. The oocytes of this kind of follicles were visibly in contrast with the surrounding granulose cells, which showed no sign of shrinkage or swelling and not an enlarged space between each other. The oocyte could contain less than 10% of vacuolization of cytoplasm. The basement membrane of the good quality follicles looked intact and attached to granulose cells with no more than 50% detachment from them. Second category follicles characterized by damages, not described in the intact follicles group. The percentage of primordial, primary, secondary and small antral follicles was also evaluated.

### Evaluation of DNA fragmentation by the TUNEL assay

The DNA fragmentation was evaluated by the immunofluorescent TUNEL technique (Merck Millipore, Germany). Briefly, after deparaffinization and rehydration of the slides, antigen retrieval was performed by using a hot water bath (95 °C) with the citrate buffer (pH 6.0) for 20 min. After cooling, tissue sections were washed D-PBS for 5 min and prepared for the enzymatic reaction by applying equilibration solution for 30 s for each section. Then slides were incubated with a 50 μL of TUNEL kit reaction mixture with terminal deoxynucleotidyl transferase (Tdt) enzyme at 37 °C for 60 min in the dark, in a humidified chamber. As the positive control, sections were treated with 10 units/mL of deoxyribonuclease I for 10 min before incubation with the TUNEL reaction mixture to induce nonspecific breaks in DNA. The negative control sections consisted of omitting the Tdt enzyme. The slides were mounted with an aqueous mounting medium for preserving the fluorescence of the tissue with Propidium iodine (Abcam Inc., USA). Follicles were considered as having fragmented DNA when oocytes were detected with a green colored nucleus. Only follicles with visible nuclei were counted. Immunostaining was evaluated using a fluorescence microscope (AxioVision, Carl Zeiss, Germany).

### Evaluation of DNA fragmentation by the TUNEL assay

The DNA fragmentation was evaluated by the immunofluorescent TUNEL technique (Merck Millipore, Germany). Briefly, after deparaffinization and rehydration of the slides, antigen unmasking was performed by using a hot water bath (95 °C) with the citrate buffer (pH 6.0) for 20 min. After cooling, tissue sections were washed D-PBS for 5 min and prepared for the enzymatic reaction by applying equilibration solution for 30 s for each section. Then slides were incubated with a 50 μL of TUNEL kit reaction mixture with terminal deoxynucleotidyl transferase (tdt) enzyme at 37 °C for 60 min in the dark, in a humidified chamber. As the positive control, sections were treated with 10 units/mL of deoxyribonuclease I for 10 min before incubation with the TUNEL reaction mixture to induce nonspecific breaks in DNA. The negative control sections consisted of omitting the tdt enzyme. The slides were mounted with an aqueous mounting medium for preserving the fluorescence of the tissue with propidium iodine (Abcam Inc., USA). Follicles were considered as having fragmented DNA when oocytes were detected with a green colored nucleus. Only follicles with nuclei were counted. Immunostaining was evaluated using a fluorescence microscope (AxioVision, Carl Zeiss, Germany).

### Ovarian follicles isolation and cryopreservation

Early-antral follicles were mechanically isolated from the slivers of ovarian cortex with the 32G sterile needles under the stereomicroscope in the flow hood prior to cryopreservation. During the isolation procedure follicles and ovarian cortex strips were kept in a saline at room temperature. After the isolation, follicles were transferred into the in-vitro culture medium (described further) for 1 h before freezing. Follicles diameter was measured prior to cryopreservation with the AxioVision software (Carl Zeiss, Germany). For cryopreservation of isolated follicles, slow freezing protocol was performed as for an ovarian tissue pieces. For the vitrification protocol 1 same cryopreservation medium was used, but with different timing: 15 and 10 min for equilibration and vitrification solutions respectively. Three variations of vitrification protocol 2 were performed with the same composition of the medium as described for the ovarian tissue slivers, but with reduction of equilibration time to 7, 7 and 5 min respectively in the described consequences of cryo solutions. As a carrier for isolated follicles in both vitrification protocols the stainless mesh with 0,045 mm aperture and 0,04 mm wire diameter was used (The mesh company, UK, Fig. 1, b).

### In-vitro follicles culture

**Fig. 2:**
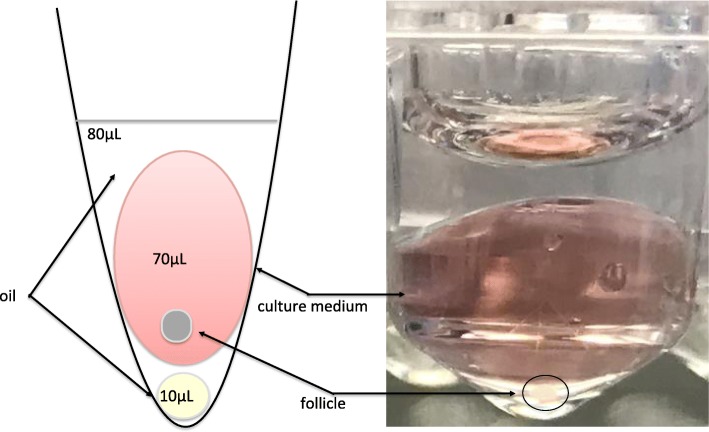
The scheme illustrating “in drop” system for in-vitro culture of isolated follicles. The scheme demonstrates V-shaped well with the 10 μL oil drop on the bottom with 70 μL drop of the culture medium, covered all together in approximately 80 μL of the oil. Additional small oil drop on the bottom ensured that follies were not attachment to the surface of the dish and were suspended in the drop of culture medium, which helped to maintain their 3D structure

Isolated follicles, assigned to one of the experimental groups were set up in the culture after being frozen/thawed individually. For the control group follicles were set up in the culture after isolation and without freezing/thawing step. Follicles were cultured in-vitro for 10 days as described by Cecconi S. and Barboni B. [33, 34]. Shortly, follicles were placed in a 70 μL drop of culture medium under the 80 μL drop of mineral oil (Fig. 2) in a 96-well dish with the V-shaped wells (Nunc, Denmark). Culture medium was TCM-199 supplemented with 50 μg/mL of ascorbic acid, 2 mM of sodium pyruvate, 2 mM of sodium glutamine, 2% FBS, 1% ITS, 1 μg/mL FSH from ovine pituitary and 1% pen/strep. Culture was carried out at 37 °C and 5% CO2 with the ambient concentration of oxygen. Half of the culture medium (35 μL) was changed every other day and stored at − 80 °C for further estradiol and progesterone determination.

### Detection of estradiol and progesterone in the culture medium

ELISA assay was performed in purpose to determine the concentrations of estradiol and progesterone in the culture media of isolated follicles. The ESLIA kits (DRG Diagnostic, Germany) were used in accordance with original instructions. Culture medium was diluted 10 times in a diluent, provided in the manufacturer’s kit.

### Viability assessment

For determination of oocyte’s viability after freezing/thawing within isolated follicles as described below, oocytes were mechanically isolated from the follicles with 32G sterile needles under the stereomicroscope in the flow hood and were left in the culture medium in incubator for one hour prior to assay. Viability was analyzed by live/dead viability/cytotoxicity kit for mammalian cells (ThermoFisher, US) according to the manufacturer instructions, with calcein AM and ethidium homodimer-1 as principle reagents. Then oocytes were mounted on the object slide and analysed under the fluorescent microscope (AxioVert, Carl Zeiss, Germany).

### Statistical analysis

Data was analyzed with the Prizm version 6 software (GraphPad Software, Inc., US). D’Agostino-Pearson omnibus test was performed as normality test. One-way ANOVA was used for analysis of variance. Differences in estradiol and progesterone production were evaluated Kruskal-Wallis test for comparison of multiple groups with Dunn’s post-test. Values with *P* < 0,05 were considered to be statistically different.

## Results

### Histological analysis of frozen-thawed ovarian tissue

**Fig. 3:**
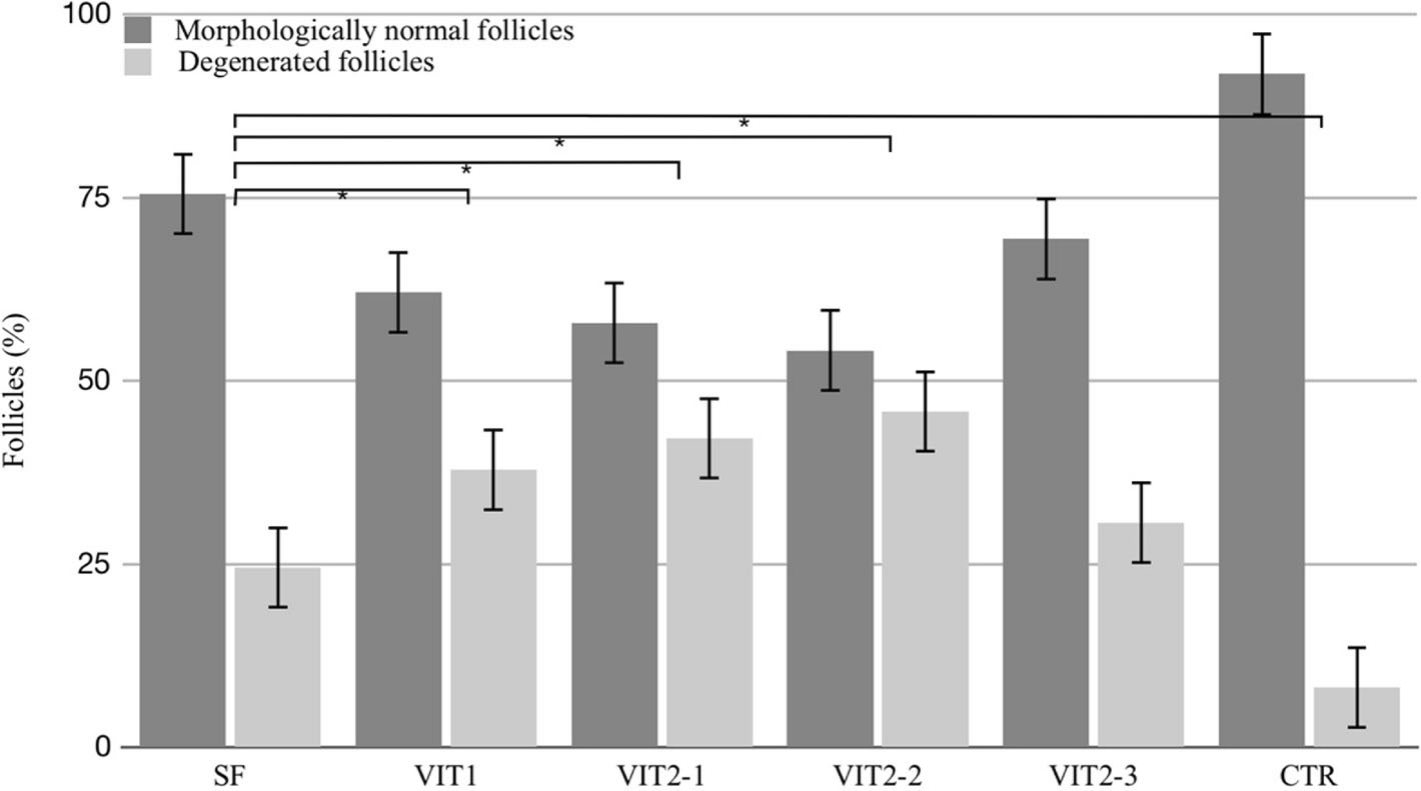
Percentage of good morphology follicles in ovarian tissue after freezing/thawing with different cryopreservation protocols. Results are % ± SE. Follicles were assessed in 5 μm sections after stating by hematoxylin and eosin. Asterisk represents significantly different results from matched protocols at *p* < 0,05

**Fig. 4:**
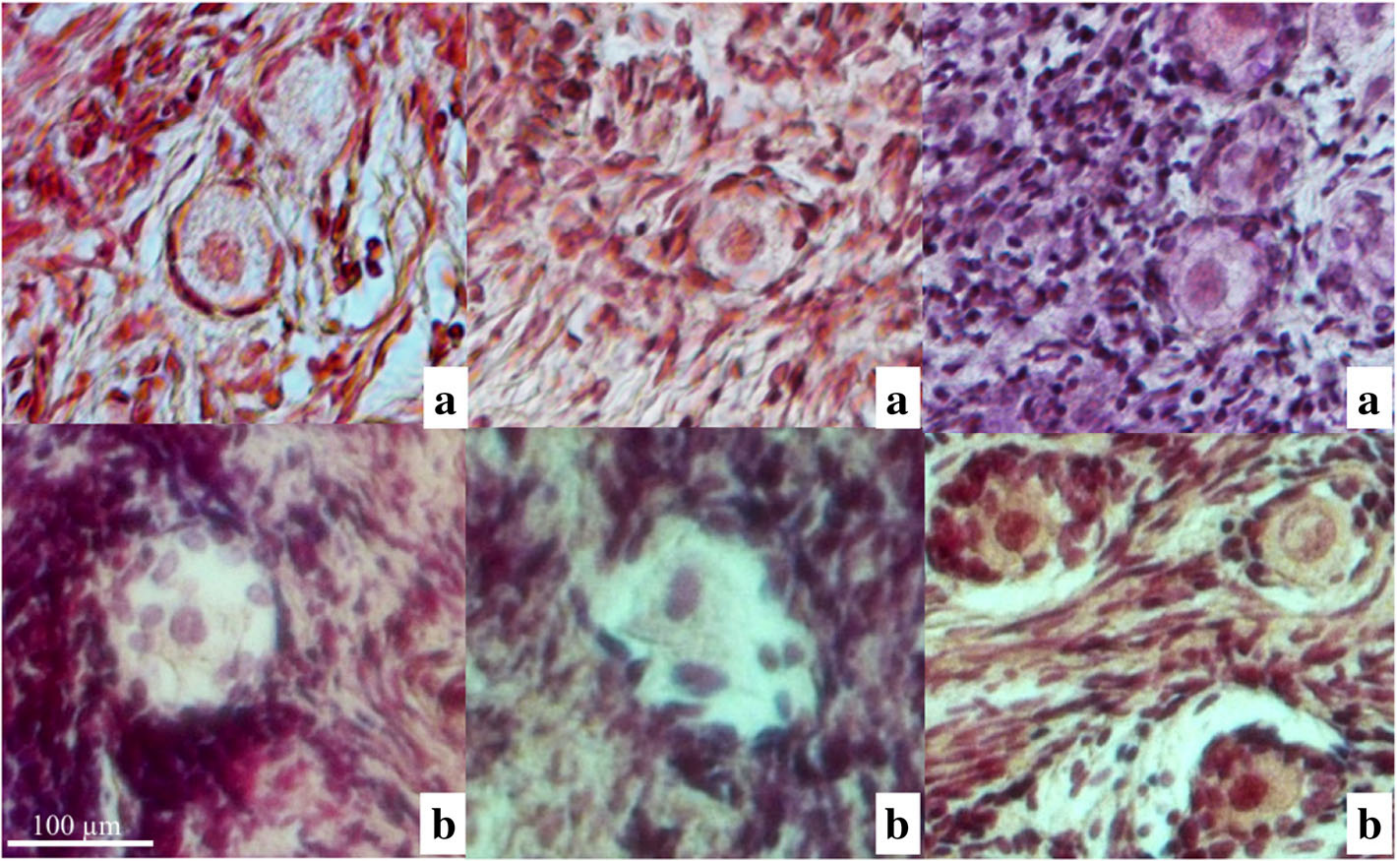
Primordial follicles morphology after cryopreservation and further warming/thawing. Primordial follicles were categorized into two quality groups: (**a)** healthy-looking follicles, (**b**) - degenerated/damaged follicles. Follicle morphology was determined by analysis of hematoxylin and eosin stained sections of ovarian cortex tissue

A total amount of 1454 follicles were counted for all of the analyzed groups together in a histological analysis of hematoxylin-eosin stained sections. This assay showed that 91,8% of all follicles in the fresh control group were of good quality. Amongst tested cryopreservation methods, slow freeing group had highest percentage of healthy-looking follicles after freezing/thawing procedures - 75,5%. As shown in Fig. 3, the percentage of morphologically normal follicles for slow freezing group was statistically different (*p* < 0,05) with another tested techniques, except the variant VIT2–3 of experimental vitrification protocol, with which it had comparable result (*p* = 0,3). The vast majority (99%) of observed follicles were in primordial stage, when primary and secondary ones were represented only by few follicles and had poor morphology. Degenerated follicles characterized by different degrees of nuclear changes such as pyknosis and nuclear fragmentation as well as shrinked cytoplasm and disorganized granulose cells (Fig. 4).

### Evaluation of DNA fragmentation by the TUNEL assay

**Fig. 5:**
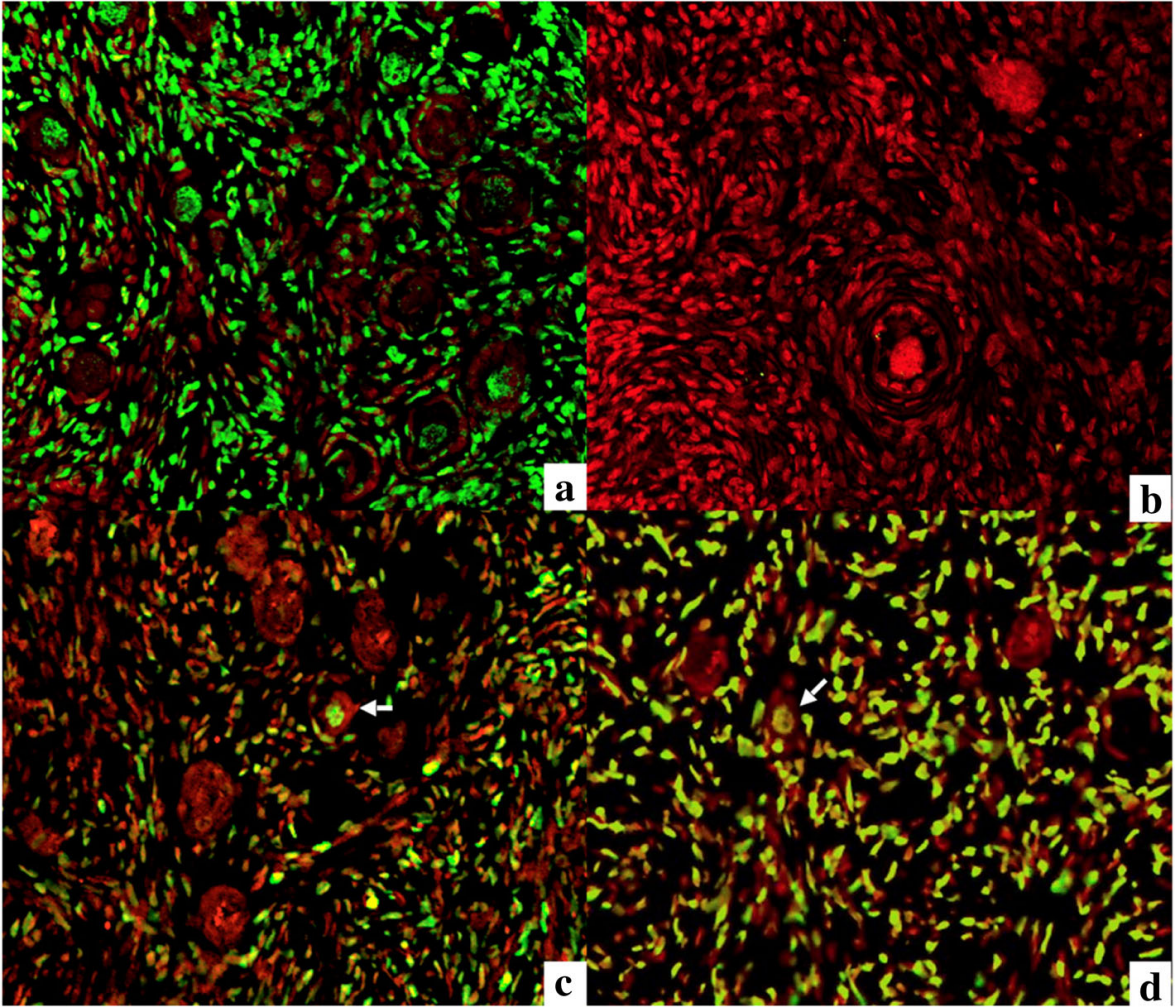
Analysis of DNA fragmentation in sections of sheep ovarian tissue using the TUNEL apoptosis detection kit. Positive control sample after the treatment with DNAse I (**a**); negative control sample with omitted terminal deoxynucleotidyl transferase enzyme (**b**); sample tissue with apoptotic follicles showed by arrows (**c** and **d**). TUNEL signal showed with green color, Propidium Iodine staining showed with red color

**Fig. 6:**
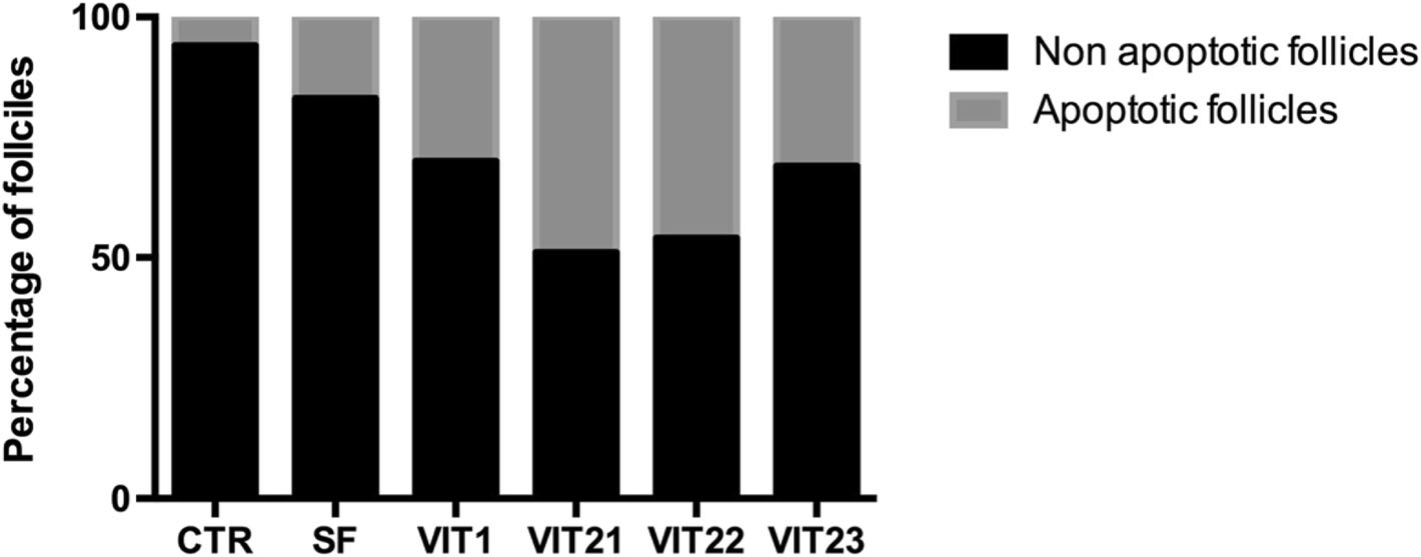
Histogram of live and apoptotic follicles distribution in the sheep ovarian cortex after cryopreservation with different protocols. Samples were analyzed by the TUNEL apoptosis detection kit. Histogram illustrates that slow freezing method ensures the better preservation of primordial follicles in the tissue, while vitrification methods 1 and 2.3 showed best results amongst tested vitrification protocols

For the analysis of DNA fragmentation, only follicles with visible oocyte nuclei were analyzed. A control for the evaluation kit was established (Fig. 5a demonstrates positive control and Fig. 5b demonstrates negative control). A positive TUNEL reaction is demonstrated for all experimental samples to different extent. Hundred of follicles were counted for each of experimental groups plus control in purpose to determine the percentage of apoptotic follicles after freezing/thawing. There were fewer apoptotic oocytes in the slow freezing group, which showed smallest proportion of apoptotic cells in the cortical ovarian tissue after freezing/tha wing (17%), when vitrification protocol 2.1 demonstrated highest percentage of apoptotic oocytes (49%). When compared to all tested cryopreservation techniques, slow freezing was the best performing technique (*p* < 0,001, Fig. 6). Amongst vitrification protocols, the method 1 and 2.3 showed fewer apoptotic follicles than other two methods (p < 0,001). In all analyzed techniques TUNEL assay indicated apoptotic damage not only to the oocytes, but also to granulose and stroma cells.

### Viability assessment of follicles after cryopreservation

**Fig. 7:**
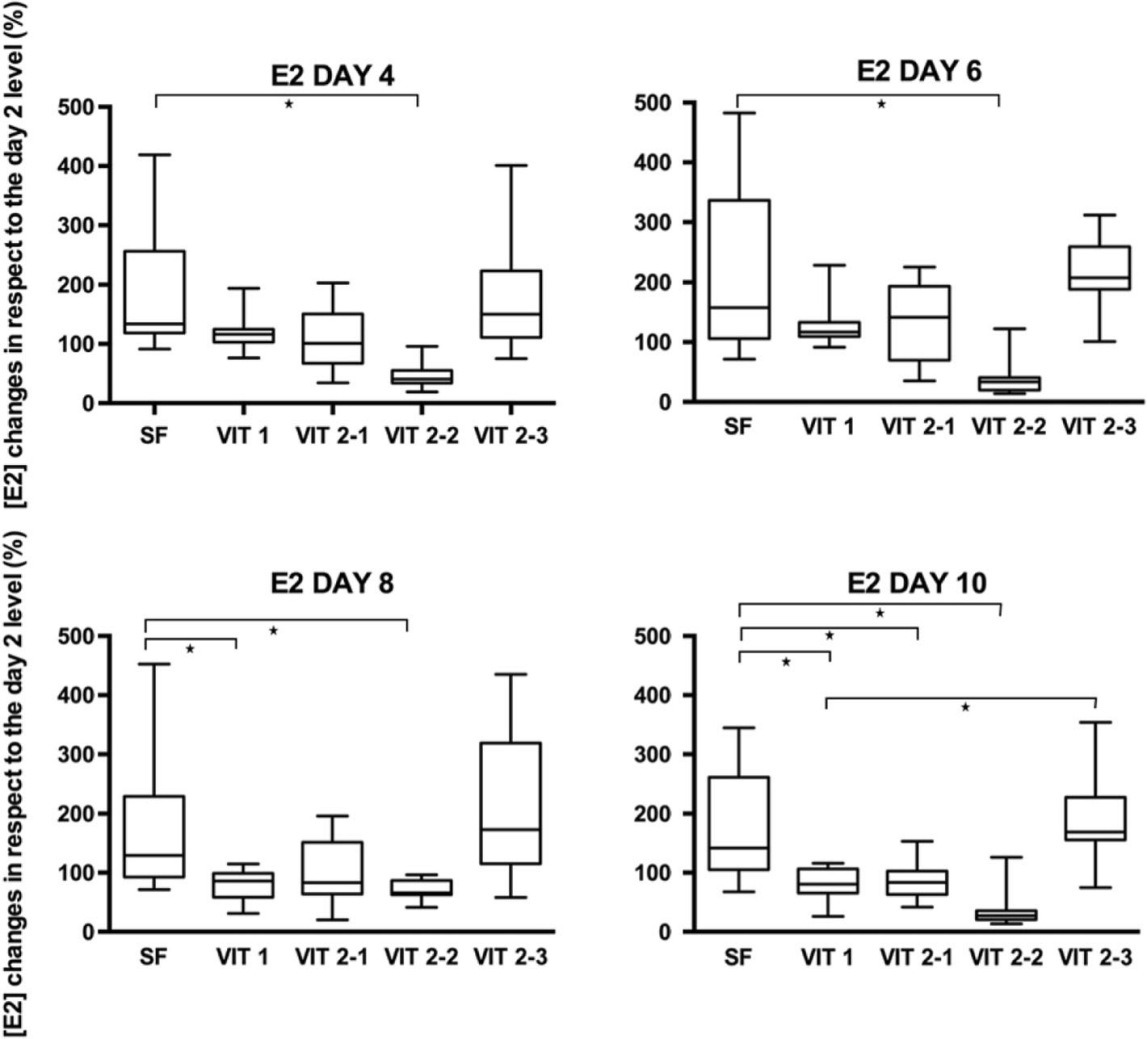
Box-plot of estradiol concentrations in different control points of the culture period. Values of estradiol concentrations in control points on day 4, 6, 8 and 10 were compared to the value on day 2 (first controlled point during the culture period) in purpose to evaluate dynamic of estradiol production. Asterisk represent significantly different results at *p* < 0.05

Twenty follicles for the each group after freezing/thawing were analyzed in purpose to determine whether oocytes remained viable or not after isolation and cryopreservation procedures (Fig. 7). Results showed that an overwhelming majority of analyzed oocytes for each experimental group remained viable after all handling procedures (Table 2). Although no statistical difference between experimental groups was found.

**Table 2:** Viability of ovine oocytes analysed by Calcein-am/Propidium iodine assay after freezing/thawing within isolated follicles

Group	*N* of viable oocytes	*N* of degenerated oocytes	Survival rate (%)
Control	20	0	100
Slow freezing	19	1	95
Vitrification 1	18	2	90
Vitrification 2–1	17	3	85
Vitrification 2–2	17	3	85
Vitrification 2–3	19	1	95

### Estradiol secretion by follicles, cultured after cryopreservation

**Fig. 8:**
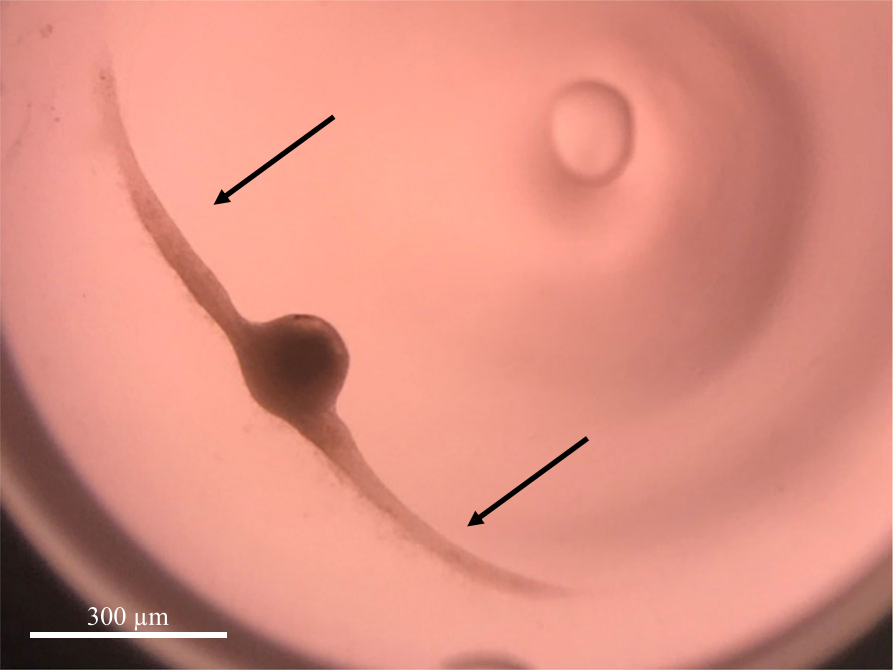
In-vitro growing follicle after 10 days of the culture. The follicle demonstrates signs of attachment to the bottom of the well (arrows) due to the external cells overgrowth, which however didn’t change normal 3D structure of the follicle

**Fig. 9:**
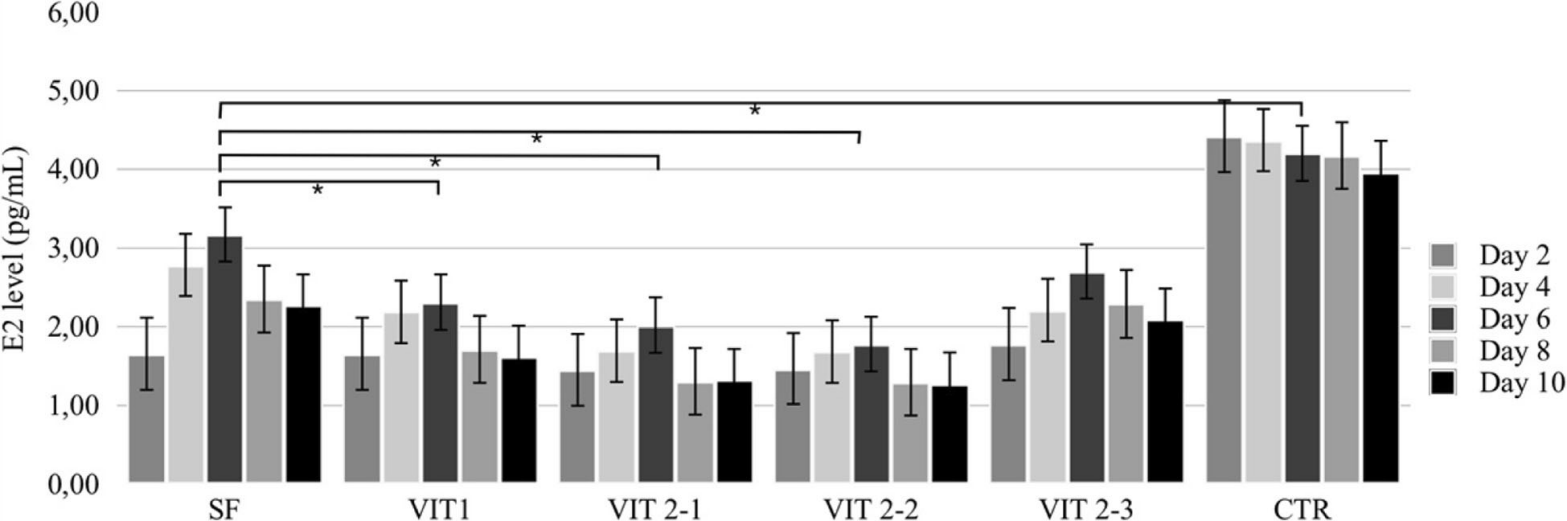
Production of estradiol by growing ovine follicles. Data are mean + SEM representing medium for 20 follicles analysed in each experimental group measured in five control points - day 2, 4, 6, 8 and 10. Asterisk represents significantly different results between matched groups at *p* < 0,05

A total number of 120 pre-antral follicles no larger than 214 μm were isolated from ovarian cortex prior to cryopreservation. Follicles were randomly allocated into 6 groups with 20 in each and cultured for 10 days. Some individual follicles from different groups were found by the end of the culture period attached to the bottom of the well, with unchanged three-dimensional organization of the follicle (Fig. 8). Follicles from the control group (Fig. 9) produced significantly higher levels of estradiol at days 4, 6, 8 and 10 when compared to another groups (*p* = 0,001). Amongst follicles that have been cryopreserved, the ones from slow freezing group on average produced more estradiol than ones from any other cryopreservation method, except protocol VIT 2–3 (p = 0,001). However, levels of estradiol appeared to decline in all groups by the end of the culture.

**Fig. 10:**
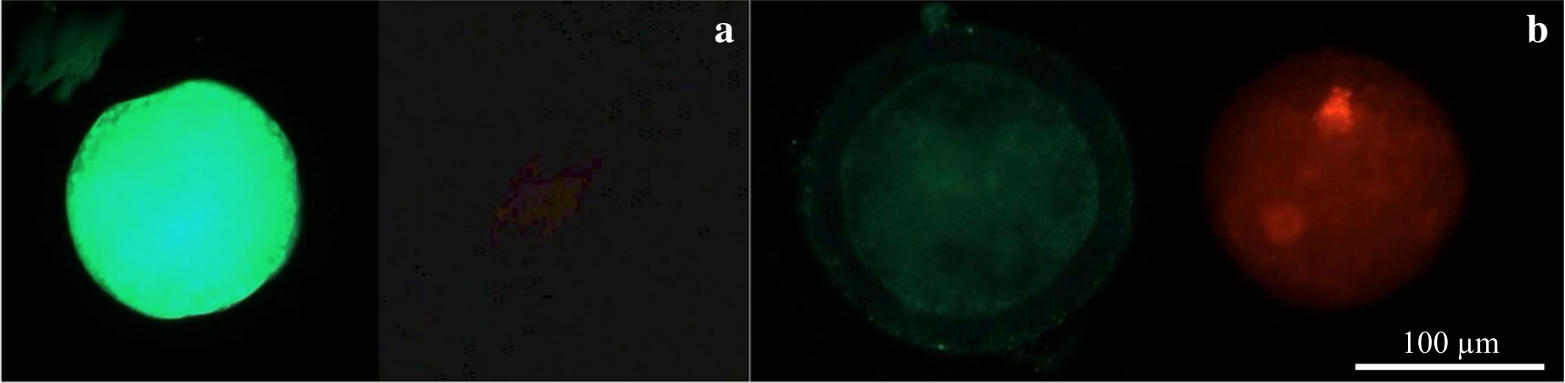
Sheep oocytes after the live-dead assay. Oocyte were derived from the follicles which were subsequently mechanically isolated, frozen and warmed/thawed. Viability of oocytes were evaluated in staining by calcein-am and ethidium homodimer-1. Letter “**a**” shows alive oocyte after freezing/thawing showed, “**b**” - degenerated oocyte

The estradiol concentrations in each of the control points (days 4, 6, 8 and 10) were compared to the concentration of the first control point - day 2 - in purpose to evaluate its dynamic during the culture period (Fig. 10). On the last day of the culture, the mean E2 level for slow freezing group was 79,6% higher its initial level on the day two and significantly different with VIT1, VIT2–1 and VIT 2–2 groups (*p* < 0,05). But the protocol VIT 2–3 had no difference with the slow freezing protocol (*p* > 0,9), thus showed better performance amongst vitrification techniques and significantly different result with reference vitrification protocol VIT1 on the final day of the culture (*p* < 0,01).

### Progesterone secretion by follicles, cultured after cryopreservation

**Fig. 11:**
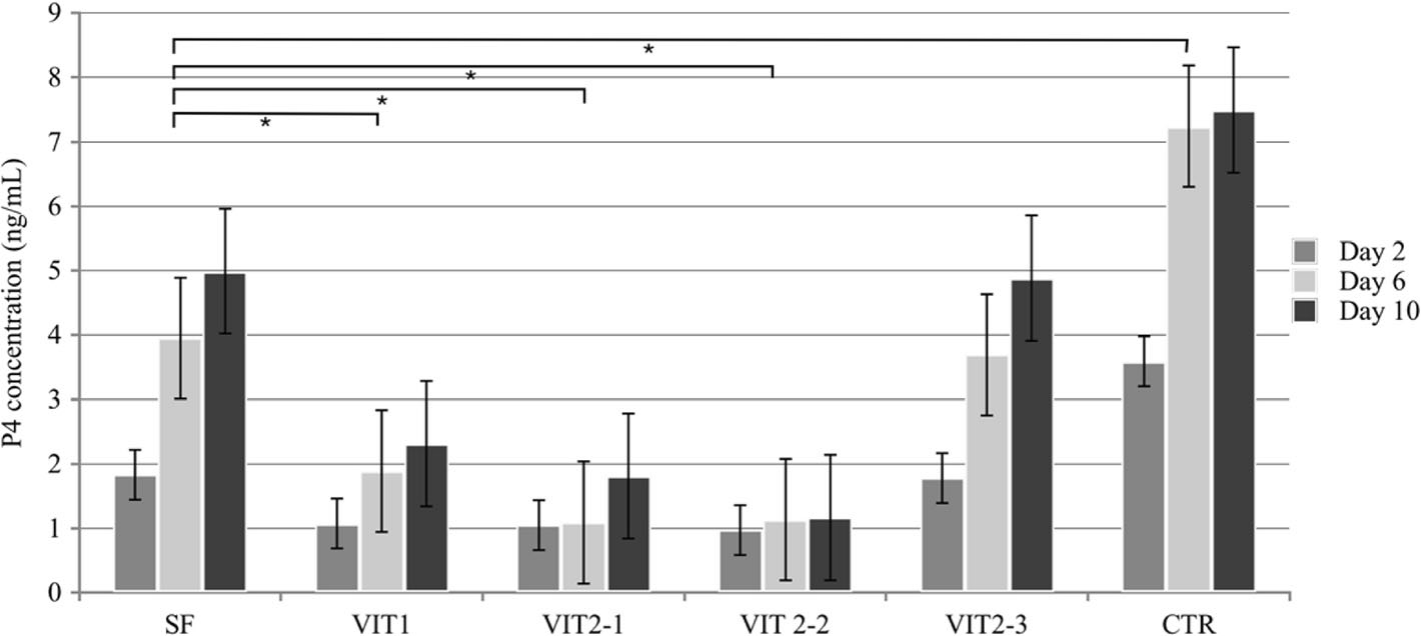
Production of progesterone by growing ovine follicles. Data are mean + SEM representing medium for 20 follicles analysed in each experimental group measured in three control points - day 2, 6 and 10. Asterisk represents significantly different results between matched groups at *p* < 0,05

In the same experimental set up as described for the estradiol secretion, the culture medium was analyzed on the presence of progesterone in three control points: day 2, 6 and 10. Overall, control group follicles produced significantly higher levels of progesterone than any experimental group (*p* < 0,001). The progesterone production increased by the end of the culture period in all groups, except for VIT 2–2. The increase in progesterone concentration during the culture period (Fig. 11) was higher for the slow freezing and VIT 2–3 groups (p < 0,01), when compared to another experimental groups, but no difference was observed when slow freezing compared to the VIT 2–3 protocol (*p* > 0,9).

**Fig. 12:**
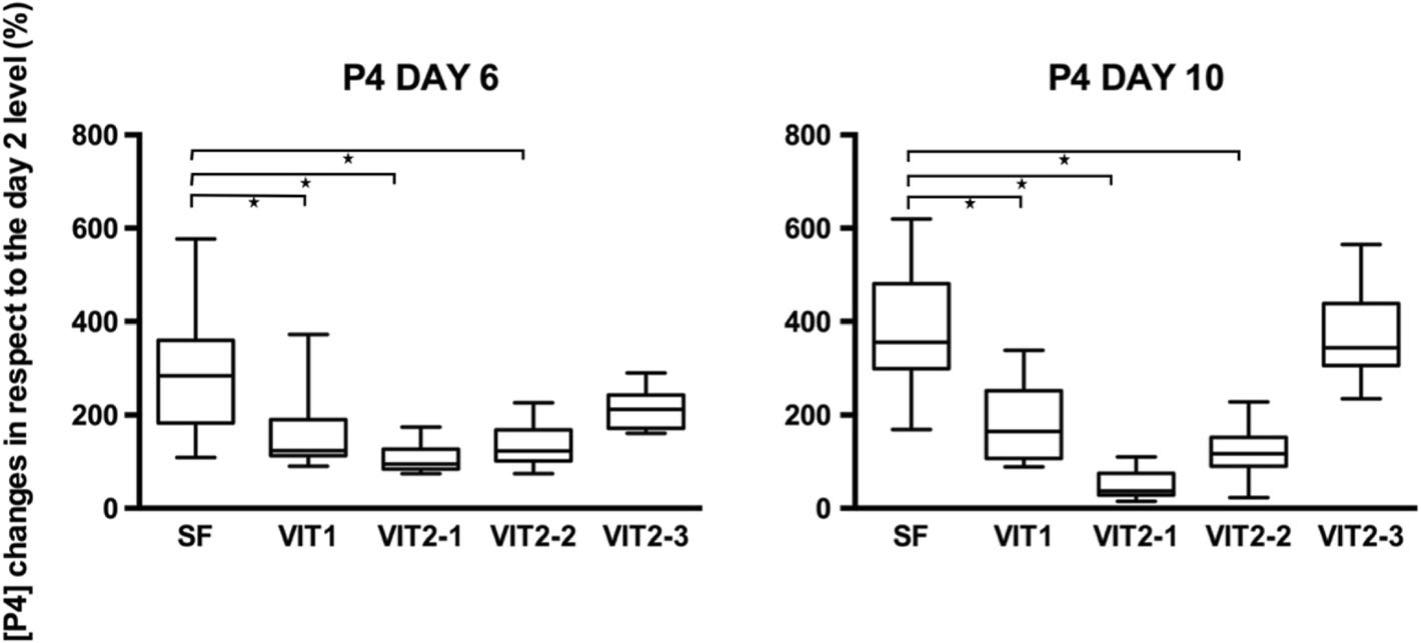
Box-plot of progesterone concentrations in different control points of the culture period. Values of progesterone concentrations in control points on day 6 and 10 were compared to the value on day 2 (first controlled point during the culture period) in purpose to evaluate dynamic of progesterone production. Asterisk represent significantly different results at *p* < 0.05

The progesterone concentration in the control points on day 6 and 10 was compared to the concentration on the day 2 in purpose to evaluate its dynamic during the culture period (Fig. 12). On the last day of the culture, the mean E2 level for slow freezing group were 3,8 times higher its initial level on the day two and significantly different with all groups (*p* < 0,05) except the VIT 2–3 protocol (*p* > 0,9).

## Discussion

In the present study, we successfully developed a vitrification protocol for ovine ovarian cortical tissue and isolated follicles using a multi-protectoral approach. Our aim was to improve cryopreservation outcomes and make survival rates after vitrification at least comparable to those after slow freezing. To increase survival rates after vitrification/thawing, we combined four cryoprotectants together instead of three in comparable vitrification protocol. These four cryoprotectants were used in lower concentrations, but cumulatively ensured sufficient total concentration of cryoprotectants in the solution. To evaluate new vitrification protocol we compared it with clinically used slow freezing method, considered as the gold standard in ovarian tissue cryopreservation [20, 35–37]. Although multi-protectoral approach helped to improve the percentage of healthy-looking follicles after freezing/thawing and as well helped to obtain higher values for steroid production by individual follicles after freezing/thawing, it did not surpass the same values for the slow freezing protocol. In the present study we used simple carrier device made of stainless steel mesh. Such type of device is inexpensive and suitable for carrying ovarian fragments or isolated follicles. The stainless steel mesh consists of criss-crossed strands of metal wires with large pores allowing drainage of excess vitrification solution. It also provides a roughened surface for the support of ovarian tissue fragments and has higher cooling rate in comparison with plastic carriers.

Most of the primordial follicles from the control group were found to have good morphology (91,8%), which was similar to the outcomes from another studies [38, 39]. When compared to any cryopreservation technique, control group had significantly more healthy-looking follicles (*p* < 0,01). That clearly indicated the impact of freezing and thawing/warming procedures and defined the main scope of our research - try to decrease as much as possible any negative changes in ovarian tissue during cryopreservation. Our results clearly indicate that follicle morphology in VIT 2–3 protocol was significantly higher than in the comparable vitrification protocol (p < 0,05). Original reports for the VIT 1 method [31, 32] did not evaluate the percentage of good quality follicles in the tissue after freezing. However they contained data on survival rates for oocytes, obtained from the follicles enclosed in the tissue after vitrification - 89%. Our results for the viability of oocytes obtained from the isolated follicles after freezing/thawing were similar to the original reports - 90%. The integrity of the basal membrane of the follicle is a major concern during freezing/thawing process, as well as nuclear quality, as they highly likely to compromise survival of the follicle. We suggest that thanks to the multi-protectoral approach and optimal combination of cryoprotectants used in VIT 2–3 protocol, morphology of the follicles after using this method was better and percentage of follicles with intact basal membrane and healthy looking nuclei was higher than in VIT 1 protocol. This is also probably because of the use of lower temperatures during equilibration steps, which helped better to preserve original quality of follicles. This results from the vitrification method 2–3 is of crucial importance, as morphological preservation of follicles is an essential condition for their further development [40].

We have supported the data on primordial follicles morphology and live/dead assay with immunostaining by TUNEL test, which appeared to be concordant. We have found significantly less apoptotic primordial follicles in the cortical tissue, vitrified by methods 1 and 2.3 (*p* < 0,001) when compared to another vitrification methods, although slow freezing group demonstrated highest number of intact follicles amongst all tested groups. We believe that the fact of low cryoprotectants concentrations, as in method 2.3, was not enough to prevent cellular damage during the freezing/warming procedure and, consequently, DNA fragmentation. It explains comparable level of apoptotic cells in the tissue with the vitrification protocol 1. But when a cell or tissue is subjected to stress conditions such as exposure to an inadequate concentration of cryoprotectants and cooling/warming procedures, as in case of method 1, the imbalance between the ROS/antioxidants can induce DNA double-strand breaks which may lead to cell apoptosis even after warming and culture/transplantation of the tissue. In such a case tissue will be affected by stress conditions in some degree even after warming and consequently the percentage of apoptotic follicles might increase even further, when for the method 2.3 it believed that lower concentrations of cryoprotectants used are less likely to affect tissue after warming.

Early-antral follicles obtained with mechanical isolation were found to have maintained their original spherical structure until the end of 10 days-long in-vitro culture with exception of four follicles which have attached to the bottom of the dish and created specific cellular strands due to external cells overgrowth (Fig. 5). That fact did not influence the estradiol and progesterone productions, however shows that in-vitro growth is better maintained when follicles are cultured being tissue-enclosed or in 3D matrix [41]. Our results confirmed that granulose cells were well preserved and follicles remained metabolically active after freezing/thawing. Estradiol and progesterone production remained highest in the slow freezing group, which says that this method is having least damaging effect among tested cryopreservation methods. Not only a higher production of estradiol in follicles after slow freezing was detected, but also a natural variability of results was observed for the protocol (Figs. 7, 9). These results correspond to the data, obtained in other laboratories on ovine ovarian tissue [42]. Amongst vitrification protocols, the variant VIT 2–3 had highest level of both steroids production and comparable to the slow freezing. After 10 days of in-vitro culture follicles from VIT 2–3 group maintained statistically higher levels of estradiol and progesterone production (*p* < 0,05), which indicates its better capacity to preserve granulose cells inside early-antral follicles than VIT 1 protocol. These results showed that after vitrification follicles have the potential to grow from pre-antral stages and with VIT-2.3 protocol their chances to grow are higher than with VIT 1 protocol. The hormonal activity of thawed and cultured follicles were selected as an evaluation criteria for effectiveness of cryopreservation protocols. Steroidogenic activity of ovarian tissue and isolated follicles is know as an indicator of tissue viability [39, 43]. In our study we found an enhanced P4 production during culture time and enhanced secretion of E2 with further decline. This correlated with results, previously obtained on steroids production evaluation on ovine granulose cells [44]. The decrease in E2 level with culture time might be due to the fact that developing early antral follicles require certain 3D surrounding, which wasn’t provided in our culture system because we didn’t aim to continue in-vitro grow of the follicles to the late stages. The production of E2 and P4 in in-vitro culture of isolated follicles indicates that the steroidogenic pathways were not disrupted after freezing/thawing.

The percentage of viable oocytes after the warming/thawing procedure was pretty high in all experimental groups (> 85% in each of the groups), however there were no statistical difference between groups (*p* > 0,5). Obtaining high viability oocytes is an absolute prerequisite for their further successful growth, either in culture or in transplantation of the frozen/thawed tissue. Thus, although we have shown no difference in oocyte survival rates between analyzed groups, it is important that most of the oocytes were found alive.

In the present study, we demonstrate that the cryopreservation of ovine ovarian tissue allows the recovery of viable primordial/primary follicles with different efficiencies according to the type of the cryopreservation technique used. So, in our attempt to improve the survival rate of the tissue or isolated follicles after freezing, we not only tried to develop new composition of the cryopreservation media, but also compared this protocol to already existing ones and determined whether there is significant difference between slow freezing and vitrification methods. Suggested vitrification solution contained four cryoprotectants: EG, PG, sucrose and ficoll, while in comparable vitrification protocol were used three: DMSO, EG and sucrose. In our protocol DMSO was replaced by PG due its high toxicity [45–47] and by considering its slower transmembrane diffusion on the bases of its molecular mass (DMSO 78.13 g/mol vs PG 76.10 g/mol). Also, DMSO is known to be an epi-mutagen, altering the DNA methylation status with methylation and demethylation in some particular loci [48]. Thus, replacement of DMSO by PG is clearly advantageous. The use of EG in our protocol was determined by its relatively low molecular mass, which correlate with the transmembrane diffusion potential (62.07 g/mol). Some previous reports also showed that EG ensures better survival rates for ovarian tissue when compared with DMSO [49–51]. Positive impact of sucrose as a non-penetrative cryoprotectant can be explained by stabilizing lipid membranes by hydrogen bonding with the polar head groups of membrane lipids [52], which is especially important under severely dehydrated conditions. Benefits of sucrose use in the freezing compositions was also described in many previous studies [53–55]. Ficoll as a highly soluble polymer acts as a non-permeable cryoprotectant by affecting the viscosity of vitrification solution thus reducing mechanical stress, coating cells, protecting the cell membrane and also preventing crystallization during freezing/thawing [56]. In comparison with another macromolecules used as cryoprotectants such as polyvinylpyrrolidone and bovine serum albumin, ficoll has advantages of lower toxicity, higher solubility and lower viscosity [57]. Ficoll has been reported previously as a reliable cryoprotectant for vitrification of murine oocytes [58], murine embryos [59], and mouse ovarian tissue [60]. Thereby, the addition of ficoll into the cryopreservation media helps in better preservation of ovine ovarian tissue from cryoinjury. Optimal combination of cryoprotectants concentrations in suggested vitrification protocol was defined by comprising three alternative solutions. After different evaluations we concluded that optimal composition, which ensured the best survival of ovine ovarian tissue, is the one with 1 M EG, 1 M PG, 1,5 M sucrose and 10% ficoll.

## Conclusion

Current study indicated that multi-protectoral vitrification solution, based on four cryoprotectants (ethylene glycol, propylene glycol, sucrose and ficoll) ensures better survival of the cortical tissue/isolated follicles, than vitrification protocol with dimethyl sulfoxide, ethylene glycol and sucrose. Moreover, equilibration with lower temperatures potentially also helped to decrease toxic effects of cryoprotectants and preserve original quality of ovarian tissue. Therefore, this method can be suggested as an improved method for the clinical cryopreservation of ovarian tissue.

## Abbreviations

DMSO: Dimethyl sulfoxide;
EG: Ethylene glycol
FBS: Foetal bovine serum
FSH: Follicle stimulation hormone
HEPES: 4-(2-hydroxyethyl)-1-piperazineethanesulfonic acid
HSA: Human serum albumin
ITS: Insulin-Transferrin-Selenium
PBS: Phosphate buffered solution
PG: propylene glycol
TCM-199: tissue culture medium-199
TUNEL: Terminal deoxynucleotidyl transferase dUTP nick end labeling
